# Intelligence test items varying in capacity demands cannot be used to test the causality of working memory capacity for fluid intelligence

**DOI:** 10.3758/s13423-021-01909-w

**Published:** 2021-04-13

**Authors:** Gidon T. Frischkorn, Klaus Oberauer

**Affiliations:** Department of Psychology, University of Zurich, Zurich, Switzerland

**Keywords:** Fluid intelligence, Working memory capacity, Capacity hypothesis

## Abstract

There is a strong relationship between fluid intelligence and working memory capacity (WMC). Yet, the cognitive mechanisms underlying this relationship remain elusive. The *capacity hypothesis* states that this relationship is due to limitations in the amount of information that can be stored and held active in working memory. Previous research aimed at testing the capacity hypothesis assumed that it implies stronger relationships of intelligence test performance with WMC for test items with higher capacity demands. The present article addresses this assumption through simulations of three theoretical models implementing the capacity hypothesis while systematically varying different psychometric variables. The results show that almost any relation between the capacity demands of items and their correlation with WMC can be obtained. Therefore, the assumption made by previous studies does not hold: The capacity hypothesis does not imply stronger correlations of WMC and intelligence test items with higher capacity demands. Items varying in capacity demands cannot be used to test the causality of WMC (or any other latent variable) for fluid intelligence.

## Introduction

Individual differences in fluid intelligence – the ability to “*reason and solve problems involving new information*” (Carpenter et al., 1990 p.404) – are strongly correlated with measures of working memory capacity (WMC; Conway & Kovacs, 2013; Kyllonen & Christal, 1990; Oberauer et al., 2005). Still, the cognitive mechanisms underlying this relationship remain elusive. One prominent account is that individual differences in WMC causally affect performance in fluid intelligence measures. This so-called *capacity hypothesis* assumes that an individual’s ability to maintain a limited amount of information active in working memory at least partly determines their performance in fluid intelligence measures (Carpenter et al., 1990; Colom et al., 2008; Unsworth et al., 2014).

The capacity hypothesis originates from an analysis by Carpenter et al. (1990) of cognitive processes associated with performance in Raven matrices tests (Raven & Raven, 2003). Their conceptual analysis yielded two aspects distinguishing high- from low-performing individuals on this fluid intelligence measure: (a) the ability to induce more abstract relations, and (b) the ability to manage larger sets of goals/rules in working memory. Whereas the first aspect is not necessarily linked to WMC, the second aspect directly maps onto WMC as a person’s ability to hold in mind a limited amount of information. Accordingly, WMC was assumed to be critical in limiting a person’s performance in Raven matrices tests.

To determine the cognitive demands of individual Raven test items, Carpenter et al. (1990) classified which rules, and how many rule tokens, an item required for a correct solution. They found that with increasing number of rule tokens required for solving an item (i.e., the capacity demands of an item) its mean error rate increased. This relationship of theoretical capacity demands and observed item difficulty (i.e., error rates) was replicated in later studies (e.g., Burgoyne et al., 2019; Little et al., 2014; Wiley et al., 2011).

On the basis of these results, previous studies claimed that the capacity hypothesis can be tested by comparing the correlation of WMC with intelligence test items of varying difficulty or capacity demands (Burgoyne et al., 2019; Little et al., 2014; Salthouse, 1993; Unsworth & Engle, 2005; Wiley et al., 2011). The underlying assumption in these studies was that more difficult items, or items with higher capacity demands, more strongly rely on WMC, and therefore should show higher correlations with measures of WMC. In detail, these studies estimated the correlation of item performance in Raven matrices (Raven & Raven, 2003) with WMC measures, such as complex span (Daneman & Carpenter, 1980) or visual arrays tasks (Luck & Vogel, 1997), to test whether this correlation increases with higher item difficulty or capacity demands. All but one study (Little et al., 2014) found no increase in correlations with WMC measures for more difficult Raven items, or for items with higher capacity demands. These results were then interpreted as evidence against a causal role of WMC for intelligence, indicating that other processes such as attention control (Burgoyne et al., 2019; Wiley et al., 2011) may be more important for intelligence differences.

A methodological critique of these types of analyses (Smoleń & Chuderski, 2015) argued that “*although intuitively attractive*” the underlying idea is “*fundamentally flawed.*” Specifically, Smoleń and Chuderski (2015) proposed that the pattern of correlations across items varying in difficulty (operationalized by mean error rates) necessarily follows a quadratic function that is tied to the amount of floor and ceiling effects for the different items. Both analytical derivations and empirical results of their study showed that, depending on the size of the correlation between two variables and the amount of floor or ceiling effects for specific items, correlations can increase, be almost stable, or decrease across items varying in their mean error rate. On this basis, Smoleń and Chuderski (2015) concluded that results from such analyses cannot be informative regarding the capacity hypothesis. These results are, however, limited to floor or ceiling effects in mean error rates, and thus do not directly reflect changes in capacity demand. Here we present a conceptual analysis of the capacity hypothesis focusing more directly on capacity demands and its implications for WMC-intelligence correlations, which nonetheless arrives at the same conclusion.

### A theoretical model of the capacity hypothesis

**Fig. 1:**
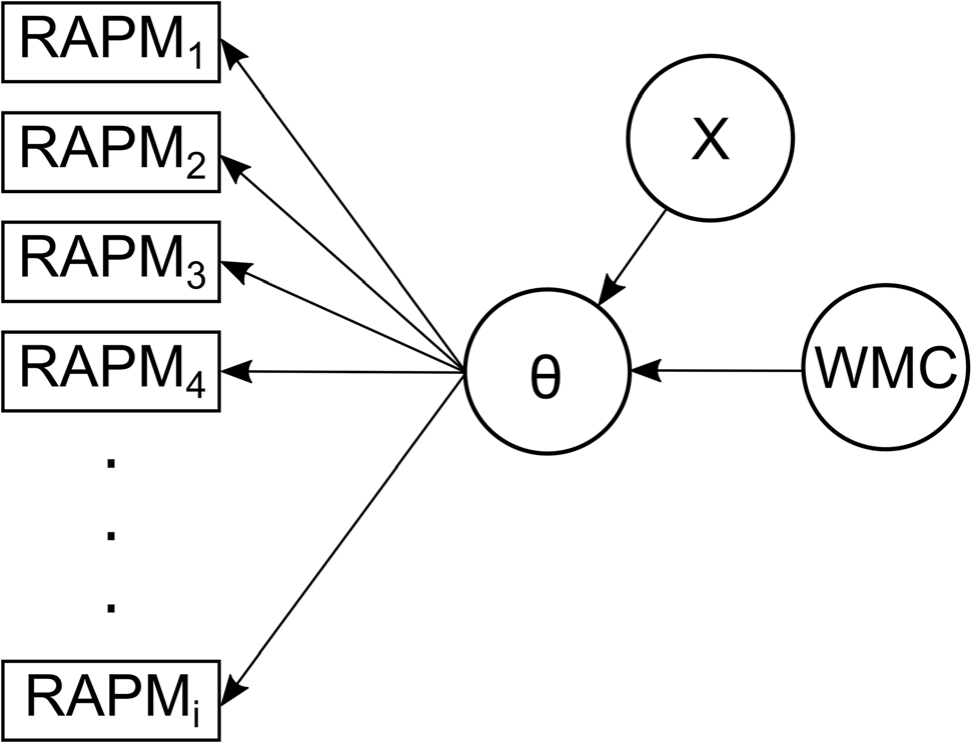
Path diagram of a theoretical model illustrating the capacity hypothesis. Working memory capacity (WMC) causally determines the latent ability (θ) that underlies performance in different intelligence test items (RAPM_1_, RAPM_2_, RAPM_3_, RAPM_4_..., RAPM_i_). In addition, other constructs (X) that are independent of WMC may also be related to the ability θ. Circles are used to illustrate latent, not directly observable constructs. Rectangles are used to illustrate manifest behavioral performance, such as the accuracy of a response in an intelligence test item

What does the capacity hypothesis actually imply for the relationship of WMC with intelligence test items of varying capacity demands? The capacity hypothesis states that WMC causally underlies individual differences in the performance of intelligence test items (see Fig. 1 for a simplified illustration of this theoretical idea). To investigate the implications of this model for the relationship between WMC and performance on different intelligence test items, item characteristics (e.g., capacity demands) of the intelligence test items can be varied systematically in a simulation to then assess how the relationship of performance on test items with WMC changes as a function of the item characteristics.

**Fig. 2:**
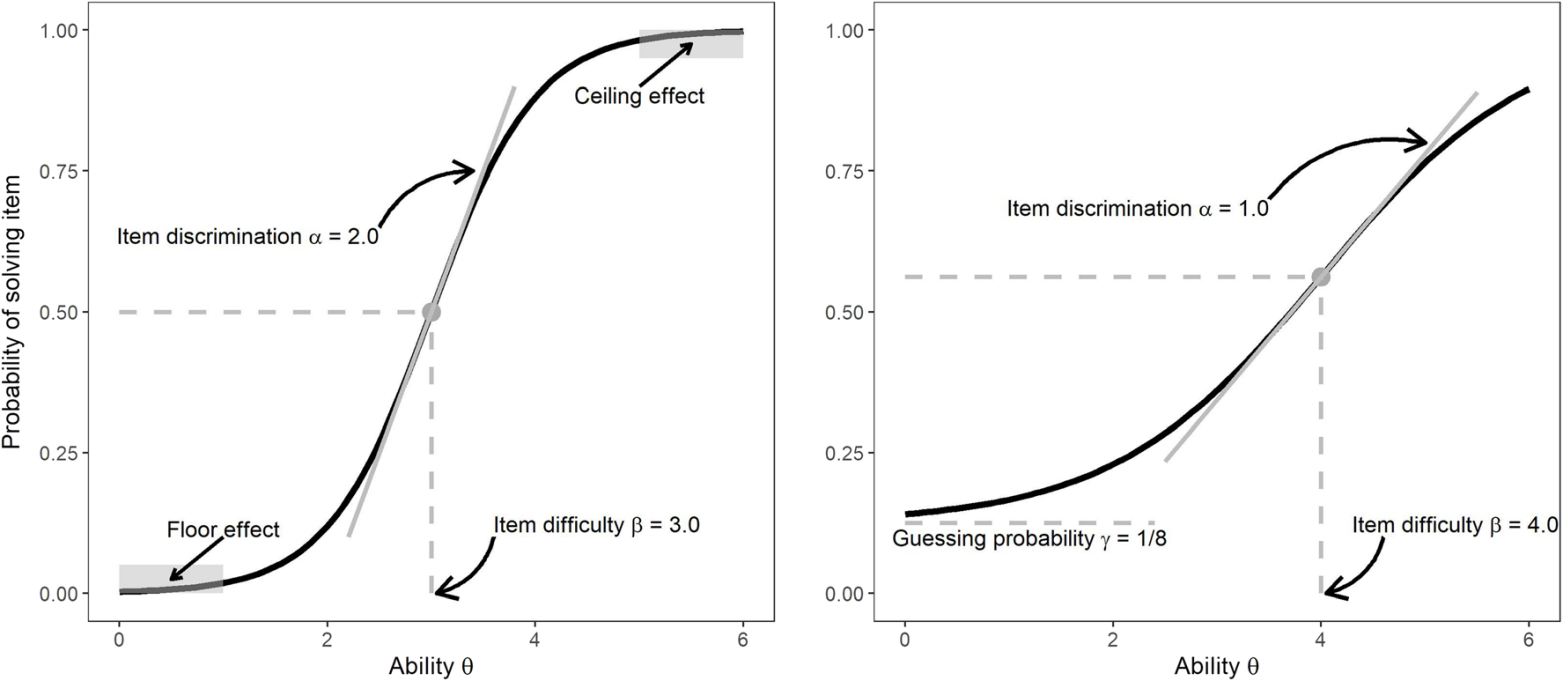
Illustration of two item-response functions (IRFs) for two items with varying difficulty β, discrimination α and guessing probability γ. The IRF on the left corresponds to an easy item with high discrimination. The IRF on the right corresponds to a difficult item with low discrimination and a guessing probability of 1/8. Although high item discrimination is better able to differentiate between people high and low in ability around its location (i.e., item difficulty), both floor and ceiling effects – as illustrated on the left side – can occur for items with high discrimination

The critical item characteristic in this scenario is arguably the capacity demand (e.g., the number of rule tokens) of an intelligence test item. The simplest and most generic way of spelling out the capacity hypothesis is to assume that if, and only if, the capacity of a person matches or surpasses the capacity demand of an item, the person should be able to solve the item. For example, an intelligence test item requiring the storage and use of three different rule tokens should only be solved by individuals with a WMC for at least three tokens. Such a deterministic threshold model is arguably unrealistic due to the inherent noisiness of mental processes. To accommodate noise, we implement a soft-threshold model, such that the probability of solving an item increases steeply as the difference between a person’s capacity and the item’s capacity demand goes from negative to positive. This relationship of available capacity and an item’s capacity demand conceptually corresponds to the relationship between ability *θ* and item difficulty or location *β* in item-response theory (IRT; Birnbaum, 1968; Rasch, 1993). As illustrated by the item-response functions in Fig. 2, the probability of solving an item increases as a function of the difference between ability *θ* and item difficulty *β.*^1^ If we interpret item difficulty *β* – as defined by IRT – as the capacity demand of an item, and the ability *θ* as a person’s capacity, this mirrors the relationship outlined above. Our model additionally incorporates the possibility that other variables besides WMC affect a person’s chance of solving an item. This could be, for instance, the person’s current alertness or motivation, or their ability to infer an abstract rule (Carpenter et al., 1990). We summarily represent such variables as *X*, and therefore model the person’s ability *θ* as the sum of WMC and *X* (see also the theoretical model illustrated in Fig. 1).

Additionally, the strength of the relationship between the ability *θ* and item difficulty *β* can be moderated by item discrimination *α* (illustrated by the slope in Fig. 2). If item discrimination is high (see left side of Fig. 2), changes in ability (e.g., from *θ* = 2.5 to *θ* = 3.5) have large effects on the probability to solve an item. In contrast, if item discrimination is low (see right side of Fig. 2), the same change in ability has a smaller effect on the probability to solve an item. The discrimination parameter can be thought of as the degree of noisiness of the soft-threshold function. Finally, the lowest possible probability to solve an item can be implemented via the guessing parameter *γ*. Formally, the probability for solving an item in such a three-parameter logistic soft threshold model is defined as:

$$ P\left(\theta \right)=\gamma +\left(1-\gamma \right)\frac{e^{\alpha \left(\theta -\beta \right)}}{1+{e}^{\alpha \left(\theta -\beta \right)}} $$

**Fig. 3:**
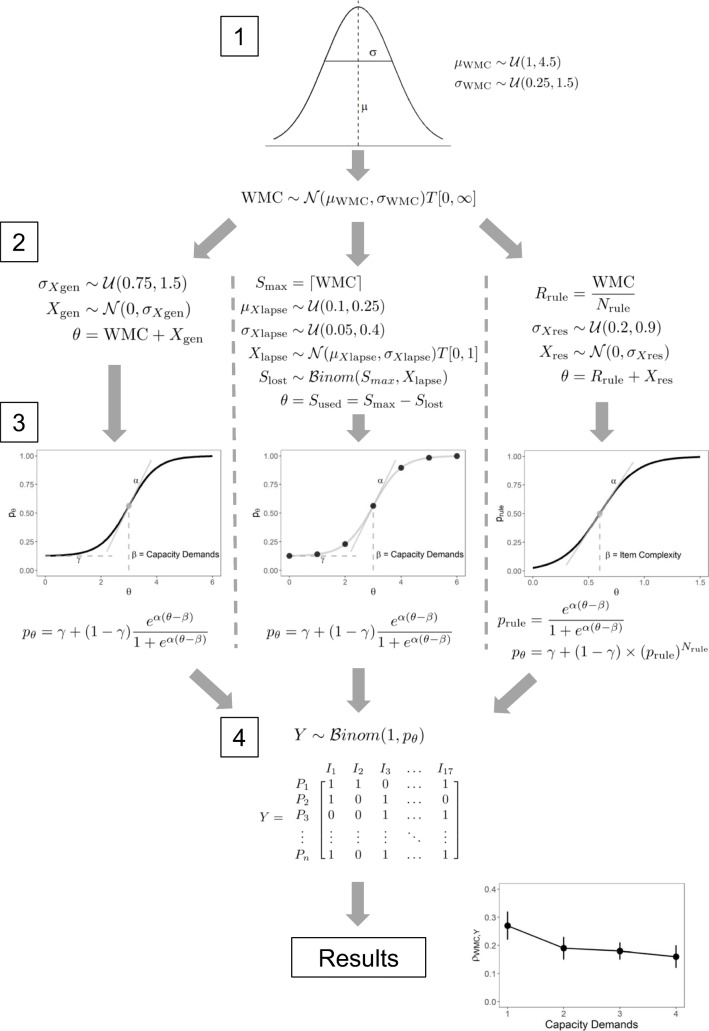
Illustration of the procedure for the simulation study. First, working memory capacity (WMC) is drawn from a normal distribution. Second, the ability underlying intelligence performance is calculated as the compound of WMC and an independent cognitive function X as specified by three different theoretical models (left: Generic, middle: Slot, right: Resource). Third, the probability of solving an item is calculated with a soft threshold model. Fourth, the performance on 17 intelligence test items (I) for each subject (P) is simulated. For the results, the correlation between item performance and WMC is calculated and plotted across capacity demands to assess the implications of the capacity hypothesis

Our implementation of the capacity hypothesis described above is very generic, making no assumption about WMC other than conceptualizing it as a continuous variable that linearly affects the ability *θ* underlying intelligence test performance*.* Some contemporary theories conceptualize WMC more specifically either as the number of discrete slots a person can use to store information in (Cowan et al., 2012; Luck & Vogel, 2013) or as a continuous resource that can be distributed across the to-be-stored representations (Ma et al., 2014). To reflect these conceptualizations of WMC, we specified two additional models that change the generic model in some respects. We assess whether these models change what the capacity hypothesis implies for the relationship of WMC and intelligence test items with varying capacity demands. All three models are specified formally in Fig. 3 and described in more detail in the *Methods* section.

### The present simulation study

Authors of several previous studies assumed that the capacity hypothesis, stating that WMC causally underlies individual differences in intelligence test performance, implies increasing correlations for items with higher capacity demands and measures of WMC. The present simulation study (see Fig. 3) implemented three different theoretical models to investigate in how far this prediction can be derived from the capacity hypothesis. This is essential to evaluate the results of previous studies and their interpretation regarding the relationship between WMC and intelligence. If the assumption motivating previous studies is true, a model implementing the capacity hypothesis should produce systematically higher correlations with WMC for items with higher capacity demands. In contrast, if this assumption is false, then a multitude of different correlation patterns across items with varying capacity demands could arise that are all in line with the capacity hypothesis.

## Methods

The R script implementing the three theoretical models, running the simulations, and generating the result plots as well as all visualizations included in this work is available at: osf.io/rt2j8.

### Procedure of the simulation study

The overall procedure of each simulation run with formal details of the three theoretical models is illustrated in Fig. 3. Each simulation run consisted of four basic steps:

1. Randomly drawing WMC for *N* = 100, 250, or 500 subjects from a Gaussian distribution. The mean and standard deviation of this Gaussian distribution were randomly drawn from uniform distributions (${\mu}_{WMC}\sim \mathcal{U}\left[\mathrm{1,4.5}\right]$; ${\sigma}_{WMC}\sim \mathcal{U}\left[\mathrm{0.25,1.5}\right]$) across each simulation run.
2. Determining the latent ability *θ* underlying intelligence test performance as composite of WMC and an independent cognitive function *X* according to one of the three theoretical models (a detailed description follows in the section *Theoretical models used for simulating performance on intelligence test items*). The standard deviation of *X* was varied to obtain different levels of correlations of WMC with the latent ability *θ* underlying intelligence test performance.
3. Determining the probability of solving an item for each subject via soft threshold functions conceptually similar to item-response functions (IRFs) from IRT. In these functions, the location parameter *β* was either specified as the capacity demand of an item as outlined in the *Introduction* (for the Generic and the Slot models), or as the complexity of an item, increasing from early to late items (for the Resource model). In addition, item discrimination was varied across four conditions: being equal, random, increasing, or decreasing across items with different capacity demands. Specifically, either one (for the equal condition) or four values (for the three remaining conditions) for item discrimination were randomly drawn ($\upalpha \sim \mathcal{U}\left[\mathrm{0.5,4}\right]$) and assigned to the four levels of capacity demand according to the condition. The guessing probability *γ* was held constant at 1/8 as Raven items force participants to choose the correct solution from eight options.
4. Using the probability of solving an item computed via the different models to simulate for each subject whether they solved the item (*Y*=1) or not (*Y*=0). To align the present simulation with the most recent publication that aimed at testing the capacity hypothesis with Raven items (Burgoyne et al., 2019), we simulated data for the 17 intelligence test items for which capacity demands were reported as the number of rule tokens they required.

We assess the implications of the simulated models for correlations of item performance with WMC across items varying in their capacity demands in two ways: First, we computed the point-biserial correlation of item performance *Y* with WMC for each item; second, we computed the Pearson correlation of item performance *Y* aggregated across items with equal capacity demands with WMC. We then assessed how these correlations changed across items with varying capacity demands. In total, we ran 1,000 simulation runs for each combination of the three different sample sizes (N = 100, 250, and 500) as well as the four conditions of item discrimination *α* (i.e., equal, random, increasing, or decreasing across capacity demands). This resulted in 12,000 simulations for each theoretical model.

### Theoretical models used for simulating performance on intelligence test items

To simulate performance on the intelligence test items as a function of WMC, we realized three theoretical models: (I) A *Generic capacity model*, conceptualizing WMC as a continuous variable that linearly translates into the ability *θ*. (II) A *Slot model* in which WMC is a discrete number of slots, each of which can hold one unit of information (e.g., one rule token). (III) A *Resource model* assuming WMC to be a continuous resource that can be equally distributed across representations needed for solving a task (e.g., across rule tokens). In addition, each of these models included an independent variable *X* to represent all additional determinants of intelligence test performance beyond WMC.

For the Generic capacity model (see left column of Fig. 3), the ability *θ* underlying intelligence test performance was calculated as the sum of WMC and *X*_gen_. Individual values for *X*_gen_ were drawn from a normal distribution with mean zero and standard deviation σ_*X*gen_. The standard deviation σ_*X*gen_ was varied across simulation runs to generate different levels of correlation between WMC and the ability *θ*: The larger σ_*X*gen_ is relative to the standard deviation of WMC (σ_WMC_), the smaller the correlation of WMC and *θ*. The probability of solving an item was obtained via a three-parameter logistic soft threshold function. For this function, the location parameter *β* was defined as the number of rule tokens an item required (*β* = 1, 2, 3, or 4) according to the analysis of Raven items by Carpenter et al. (1990). As explained in the introduction, the location *β* of this function can reasonably represent the capacity demand of an item in this model of the capacity hypothesis. In addition, item discrimination *α* could be equal, random, increasing, or decreasing across item difficulties. The guessing probability *γ* was held constant at 1/8 for all simulation runs as Raven items force participants to choose the correct solution from eight options. Using these parameter settings and the ability *θ* calculated from WMC and *X*_gen_, the probability for solving each of the 17 items was calculated. Finally, the probability for each subject and item was used to simulate which Raven items a subject was able to solve by drawing from a Bernoulli distribution.

For the *Slot model* (see middle of Fig. 3), the ability *θ* was calculated as the maximum number of slots available for a person (S_max_) minus the number of slots lost due to attentional lapses (S_lost_; for a similar implementation see Adam et al., 2015). The maximum number of slots for each person was determined as the continuous WMC rounded up to the next integer. The number of slots lost due to attentional lapses was drawn from a Binomial distribution with S_max_ draws and the probability for an attentional lapse *X*_lapse_. Individual values for *X*_lapse_ were drawn from a normal distribution truncated at zero and one, with mean and standard deviation varying across simulation runs. Again, the standard deviation of *X*_lapse_ was varied to achieve different levels of correlation between WMC and the ability *θ*. As in the Generic model, the probability of solving an item was obtained via a three-parameter soft threshold function. The location *β* of this function was again specified as the number of rule tokens required by an item, and item discrimination *α* being equal, random, increasing, or decreasing across items with varying capacity demands. Like in the generic capacity model, the probability for solving an item obtained via this soft threshold function was used to simulate the performance on each of the 17 intelligence test items via a Bernoulli distribution.

For the *Resource model* (see right side of Fig. 3), a continuous WMC resource was divided equally among the rule tokens required by an item, so that with higher capacity demand, less of the resource was assigned to each rule token. The ability to successfully apply each rule token *θ* was the sum of the resource assigned to that token and an independent cognitive function *X*_res_. Conceptually, *X*_res_ can be understood as an additional ability to apply or use a rule. Unlike for the other two models, the ability *θ* was not directly transformed into the probability of solving an item, but instead it determined the probability of successfully maintaining and applying each rule token (*p*_rule_). To obtain this probability, we again used a three-parameter soft threshold function. The location *β* of this function represented the difficulty of encoding and applying an individual rule independent of the capacity demands. We assumed *β* to increase linearly from the first to the last item. This was done to reflect that the kind of rules get more complex, and figural representation more abstract, from early to late Raven items (Carpenter et al., 1990). Specifically, the first item required few resources (*β* = 0.4) per rule, whereas the last item required substantial resources (*β =* 0.8) per rule. Item discrimination *α* was varied the same way as in the Generic and Slot models, but guessing probability was set to zero, as this function does not represent the probability of solving an item, but of successfully applying a single rule token. To calculate the probability of solving an item, *p*_rule_ was multiplied by itself as often as there were rule tokens for the item, reflecting the fact that all rule tokens need to succeed to successfully solve an item. To account for the guessing probability, the probability for solving an item was scaled to be in the range between 1/8 to 1. As in the previous models, the resulting probability of solving an item was used to randomly draw from a Bernoulli distribution to simulate the performance on 17 intelligence test items with varying capacity demands.

Together, these three models cover different theoretical conceptualization of WMC. In combination, they assess whether conceptualizing item difficulty *β* as the number of rule tokens an item requires (Generic and Slot model), or the capacity demand of applying individual rules (Resource model) changes the implications of the capacity hypothesis. Moreover, they test whether implementing different conceptualizations of WMC, as well as of variable *X* that additionally influences intelligence, affects the correlation of item performance with WMC across varying capacity demands.

### Evaluation of simulation results

We z-transformed the point-biserial correlations of item performance with WMC and aggregated them across items requiring the same number of rules. We then back-transformed these aggregated correlations to correlation coefficients and plotted them across the capacity demands (i.e., the number of rule tokens) of an intelligence test item. Pearson-correlations did not need to be aggregated as they were computed for the aggregated performance on intelligence items with equal capacity demands, so we directly plotted them as a function of the capacity demands. For the Generic and Slot models, this procedure is equal to plotting the correlations across the location parameter *β* as implemented in the soft threshold functions. For the Resource model, rule difficulty *β* varied independently of the capacity demands of an item; the capacity demand has its effect through dividing WMC by the number of rules.

The main question is whether correlations of item performance with WMC consistently increased with the capacity demands of items, as some authors have assumed would be predicted by the capacity hypothesis. We answer this question based on the systematic trends visible in the plots. As the precision of these trends can be arbitrarily increased through running more simulations, inference statistics are neither warranted nor needed for evaluating these trends.

## Results

**Fig. 4:**
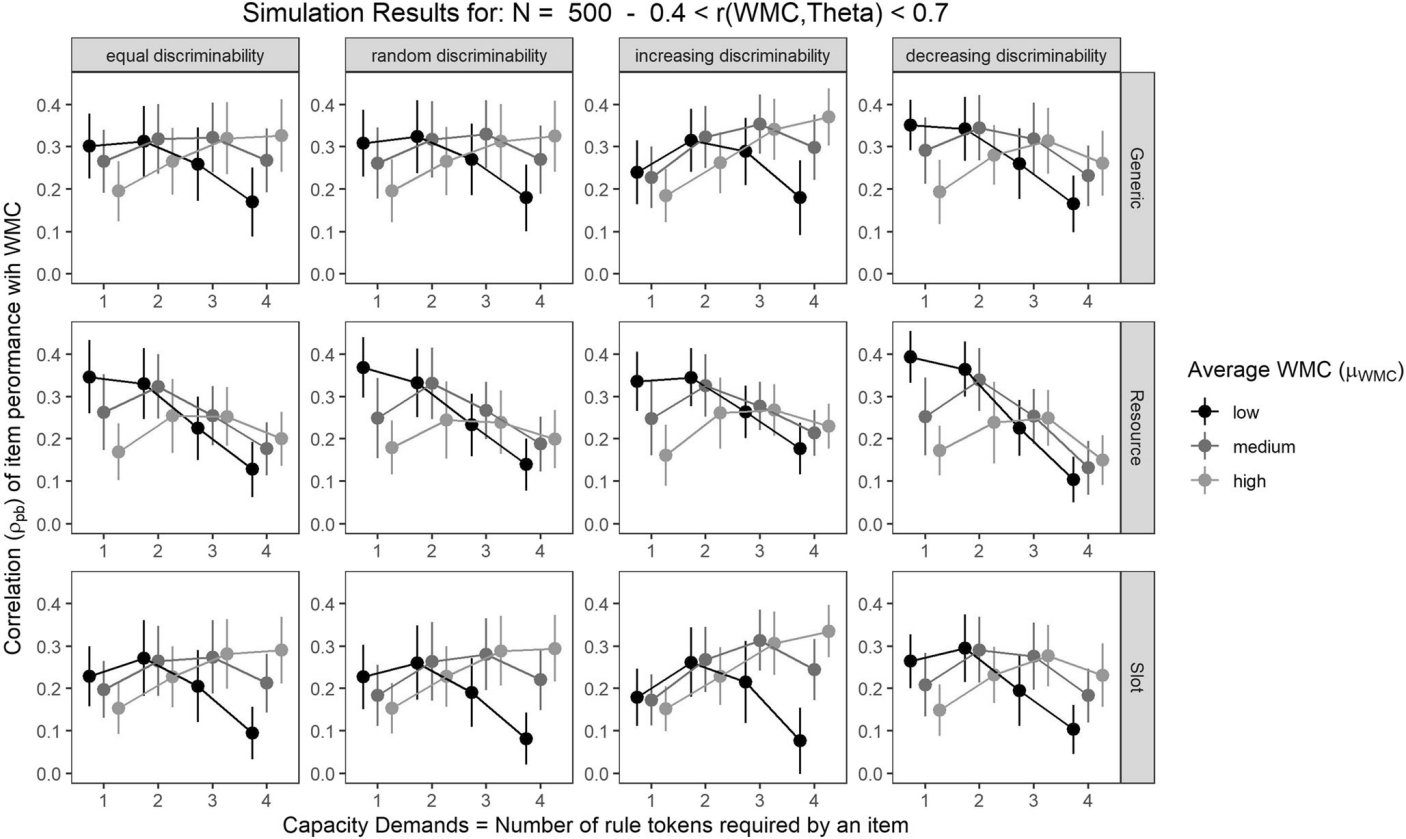
Pattern of point-biserial correlations averaged across the capacity demands of an item for the simulations with sample size N = 500 and the correlation of WMC and the ability θ underlying intelligence test performance in a medium range (0.4 < r < 0.7). The rows separate results from the three different theoretical models, the columns separate results for different patterns of item discrimination across item difficulty

Figure 4 illustrates the pattern of aggregated point-biserial correlations of item performance with WMC across items varying in capacity demands (*N* = 500, 0.4 < *r*_WMC,θ_ < 0.7).^2^ Although all three theoretical models (shown in the different rows) assumed that item performance depends on the relation between a person’s capacity and an item’s capacity demand, the pattern of correlations of item performance with WMC does not consistenly increase for items with higher capacity demands. On the contrary, the results indicate that the correlations can decrease, increase, or remain fairly constant across items varying in their capacity demands.

Specifically, the pattern of correlations across items varying in capacity demands depended on the mean level of WMC in the simulated samples and the theoretical model used to simulate the capacity hypothesis. The Generic and Slot models provide similar results: The pattern of correlations across items with varying capacity demands changes with the mean level of WMC in the simulated samples. In low WMC samples (illustrated by the black dots and lines), correlations of WMC with item performance decrease with increasing capacity demands, whereas in high WMC samples (illustrated by the light gray dots and lines), correlations increase with increasing capacity demands. In the Resource model, correlations of WMC with item performance generally tended to decrease with increasing capacity demands. This pattern was mitigated in high WMC samples, trending towards a quadratic pattern. However, correlations for items with the highest capacity demands never surpassed correlations for items with medium capacity demands.

**Fig. 5:**
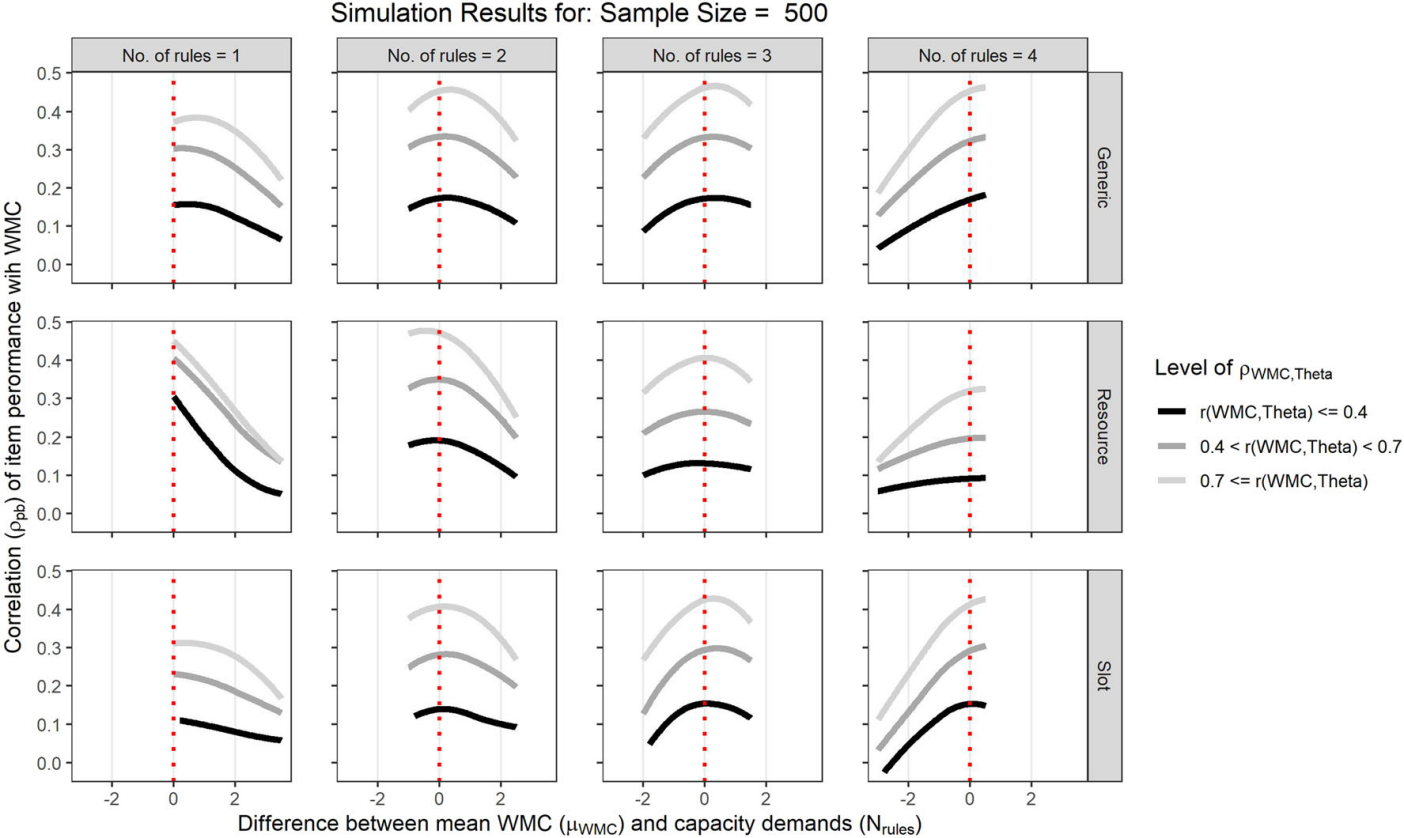
Correlation of item performance with WMC plotted depending on the difference of mean working memory capacity (WMC) and the capacity demands of an item. The vertical dotted red line indicates a perfect match between mean WMC in the sample and capacity demands of an item (i.e., μ_WMC_ - N_rules_ = 0), and the shaded lines represents the average pattern of correlations for different levels of correlations between WMC and the ability θ underlying intelligence test performance fitted with a locally weighted least squares regression

It appears that the mean level of WMC in a sample relative to the capacity demand of an item determines the pattern of correlations. To bring out this general trend more clearly, we plotted the correlation of item performance with WMC as a function of the difference between average WMC in the sample and the capacity demand of an item. Figure 5 clearly illustrates that the correlation of item performance with WMC is maximal when the average WMC in the sample matches the capacity demand of an item (i.e. μ_WMC_ - *N*_rules_ = 0) independent of its absolute capacity demand (illustrated by the different colums). Critically, this is also true for the Resource model (middle row) in which rule difficulty *β* was independent of the capacity demand of an item. In addition, the line shading in this plot illustrates that the correlation of item performance with WMC increases the stronger WMC is correlated with the ability *θ* underlying intelligence test performance.^3^

**Fig. 6:**
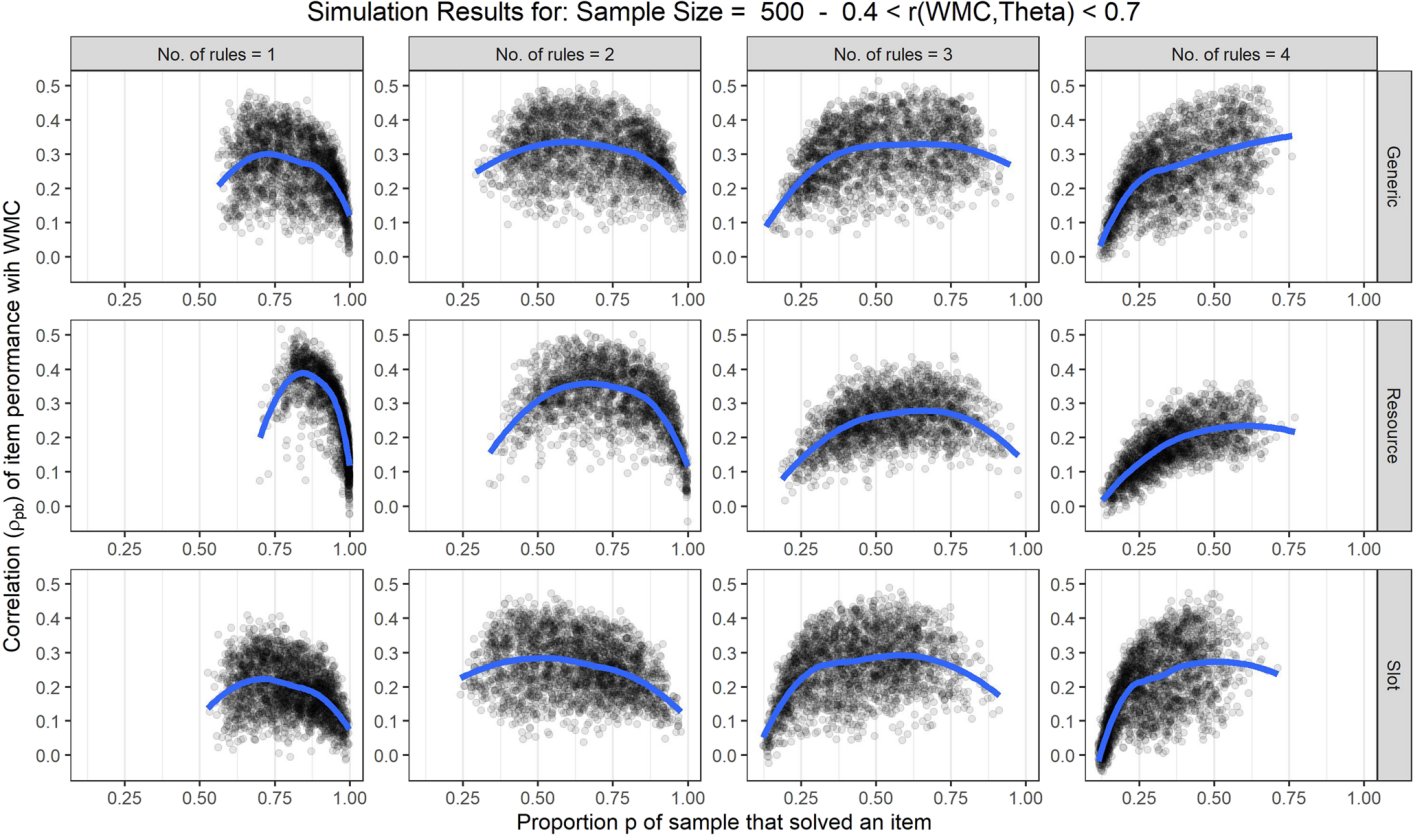
Point-biserial correlations of item performance and working memory capacity (WMC) plotted against the proportion of subjects that were able to solve an item. The shaded dots represent the single correlations estimated for each item, the blue line indicates the general pattern estimated via locally weighted least squares regression models. In addition, the different columns separate items with varying capacity demands, and the three rows illustrate results for the three different theoretical models

Finally, we compared our results to the results of Smoleń and Chuderski (2015) indicating that the correlation of item performance with WMC follows a quadratic pattern due to floor and ceiling effects. We plotted the correlations as a function of the proportion of simulated subjects that were able to solve an item (i.e., the item difficulty *p* in classical test theory). Figure 6 shows that for all three conceptual models of the capacity hypothesis (shown in the different rows) the results of our simulations replicated the result of Smoleń and Chuderski (2015): Correlations follow a quadratic pattern across varying item difficulty *p*. However, in addition to floor or ceiling effects determining the quadratic pattern of correlations, for items with low capacity demands (columns on the left in Fig. 6) correlations decreased before reaching floor (i.e., guessing probability = 1/8). This shows that it is not solely the restriction of variance by floor and ceiling that determines the quadratic pattern of correlations.

In sum, the results from this simulation show that assuming any of three different theoretical models in line with the capacity hypothesis does not imply that correlations with WMC increase with the capacity demands of intelligence test items. Instead, the degree to which mean WMC in the sample matches the capacity demands of an item determines the correlation of item performance with WMC. In addition, the correlation of item performance with WMC increases as the correlation of WMC with the ability θ underlying intelligence test performance in the sample increases. Taken together, these effects result in increasing, decreasing, or constant patterns of correlations with WMC across items with varying capacity demands. Therefore, contrary to the assumptions underlying previous studies, the capacity hypothesis does not imply that correlations between items with higher capacity demands and WMC should be larger than between items with low capacity demands and WMC.

## Discussion

The aim of the presented simulation study was to assess what the capacity hypothesis implies for the pattern of correlation of items varying in their capacity demands with WMC. We implemented three different theoretical models of the capacity hypothesis and simulated data to assess what these models imply for the pattern of correlations between WMC and item performance across varying capacity demands. Unlike what authors of previous studies assumed (Burgoyne et al., 2019; Little et al., 2014; Salthouse, 1993; Unsworth & Engle, 2005; Wiley et al., 2011), increases in the capacity demands of an item did not imply an increase in correlation between item performance and WMC. Instead, the results of the present simulation study show that the correlation of item performance with WMC depends on two variables: The proportion of variance that WMC contributes to the ability *θ* underlying intelligence test performance, and the degree to which an item’s capacity demand matches the sample’s mean WMC.

We acknowledge that our three models do not exhaust all possible implementations of the capacity hypothesis. Yet, they are sufficient to demonstrate that there are several reasonable implementations of the capacity hypothesis that do not imply larger correlations of WMC with item performance as capacity demands increase. Even if there were other implementations of the capacity hypothesis that do imply consistently increasing correlations, this prediction would still not follow from the capacity hypothesis per se, but only from one particular version of it. In sum, the conclusions from our conceptual analysis of the capacity hypothesis converges with the statistical critique of (Smoleń & Chuderski, 2015): The pattern of correlation of item performance with WMC across items varying in their capacity demands bear no information regarding the causality of WMC for intelligence differences.

### Do previous results and the presented simulation confirm the capacity hypothesis?

It would be premature to conclude from the presented simulation that results of previous studies confirmed the capacity hypothesis. Rather, the results of previous studies are consistent with the capacity hypothesis, and hence do not falsify it. In fact, the capacity hypothesis does not appear to make specific predictions regarding the pattern of correlations across items varying in difficulty and WMC. Therefore, the conclusion drawn by previous studies of this pattern (Burgoyne et al., 2019; Unsworth & Engle, 2005; Wiley et al., 2011), that not WMC but other cognitive processes such as attention control are more relevant for intelligence differences is not warranted.^4^

The present simulation results are not limited to WMC as one candidate cognitive function underlying intelligence differences. The results hold for any cognitive function, such as attention control or speed of information processing, assumed to determine the latent ability *θ* underlying performance differences on intelligence test items. Therefore, the analysis of correlation patterns across intelligence test items of varying capacity demands with any indicator of a hypothetical cause of intelligence will not be informative regarding its causality. Instead more elaborate and theoretically grounded measures for specific cognitive processes (Frischkorn & Schubert, 2018) or experimental studies that use manipulations that ideally target a single cognitive process (Rao & Baddeley, 2013; Schubert et al., 2018) are needed to investigate which cognitive processes causally underlie intelligence differences.

## Footnotes

1 Please note that although item difficulty or location *β* is interpreted as item difficulty for item-response functions used in IRT, it is not to be confused with item difficulty *p* as defined by classical test theory. Other than in CTT, *β* does not represent the proportion of people that were able to solve an item.

2 Results plots for smaller sample sizes (N = 100, 250) and different levels of *r*_WMC,θ_ , as well as results for the Pearson correlations can be found online: osf.io/rt2j8. Specifically, plots for all sample sizes can be found in the “Figures” folder. In general, the result patterns were highly consistent regarding the ordinal pattern of correlations for point-biserial and Pearson correlations (average consistency across the 1,000 simulations for the different models and conditions: .91 < *r* < .98). In addition, correlation estimates were higher overall for Pearson correlations: the average Pearson correlation across the 1,000 simulation runs for the different models and conditions was .33 < *r* < .41, compared to .23 < r < .29 for point-biserial correlations.

3 This correlation depended on the ratio between variability in WMC and the variability in X.

4 Other empirical studies analyzing correlations between measures of WMC, attentional control, and intelligence provide inconsistent results on this research question. Specifically, some studies indicated an important role of attentional control (Draheim et al., 2021), whereas other studies indicated only small contributions from attentional control (Frischkorn et al., 2019; Rey-Mermet et al., 2019). For a broad review on this topic see von Bastian et al. (2020).
